# A Scoping Review on Fungus and Mycotoxin Studies in the Building's Environment: Mycotoxin Analysis by Mass Spectrometry

**DOI:** 10.1155/2024/8581029

**Published:** 2024-01-27

**Authors:** Salina Abdul Rahman, Nurul Izzah Ahmad, Roshan Jahn Mohd Salim, Nur Jannaim Muhamad, Anis Syuhada Omar Hamdan, Yin-Hui Leong

**Affiliations:** ^1^Institute for Medical Research, National Institutes of Health, Ministry of Health Malaysia, No. 1, Jalan Setia Murni U13/52, Shah Alam 40170, Selangor, Malaysia; ^2^National Poison Centre of Malaysia, Universiti Sains Malaysia, Georgetown 11800, Penang, Malaysia

## Abstract

It has been well-established that mycotoxins are poisonous chemical metabolites secreted by certain molds. Some of them significantly affect the health of humans and livestock. Increasing attention is now being paid to uncovering and identifying mycotoxins' presence in the building's environment. However, the main challenge remains in suitable and reliable analytical methods for their identification and detection in infected structures. GC-MS and LC-MS/MS techniques have been used extensively for mycotoxin analysis, and advancement in these techniques enabled a more comprehensive range of mycotoxins to be detected. As such, this study aimed to address a brief overview of various phenomena of existing sample collection, preparation, and analysis to detect mycotoxins in the building's environment. This scoping review includes articles from 2010 to 2020 available from PubMed, Scopus, Cochrane, Wiley, Google Scholar, and ScienceDirect. Duplicate articles were removed, and exclusion criteria were applied to eliminate unrelated studies, resulting in 14 eligible articles. The present study provides an overview of mycotoxin analysis by GC-MS and LC-MS/MS in buildings. Many techniques are available for analyzing and detecting multiple mycotoxins using these methods. Future efforts would focus on rapid assays and tools enabling measuring a broader range of mycotoxins in a single matrix and lower detection limits. In addition, it would assist future findings on new techniques and mycotoxins that existed in the building's environment.

## 1. Introduction

Mycotoxins are secondary metabolites of fungi associated with various toxicities in humans and animals. They have long been studied because of their extensive exposure to food and feed commodities and their potential use as therapeutic drugs and biological warfare agents. Due to the prevalence of mycotoxin contamination in foods and feeds, many years of research have focused on ingesting these toxic compounds. Inhalation of mycotoxins has yet to attract much attention, and available reports point mainly to occupational and agricultural settings. In the 2000s, mycotoxins were hinted to be toxic agents adverse to human health through inhalation exposure in a nonagricultural indoor environment. Airborne mycotoxins have been reported in moldy buildings other than agricultural settings [1, 2]. They originate from the fungal pollution of the indoor environment, e.g., sterigmatocystin and aflatoxins produced mainly by *Aspergillus* spp. which include *A. versicolor* and *A. flavus*, and macrocyclic trichothecenes produced by *Stachybotrys chartarum*. A comprehensive review of fungal pollution in an indoor environment was documented by Khan and Karuppayil [3]. Wood or wood-based products are susceptible to infestation by *Cladosporium* and *Penicillium* (*Penicillium brevicompactum* and *Penicillium expansum*), *Trichoderma*, and *Aspergillus* [4, 5]. *Paecilomyces variotii*, *Trichoderma harzianum*, and *Penicillium* species attack polyurethanes used in composites for insulation [6]. Also, dust on the surfaces and inner wall materials used in buildings, such as prefabricated gypsum board, paper, and glue, represents an excellent substrate for fungal growth. According to D'Mello [7] and Ciegler and Bennett [8], one mold species can produce several mycotoxins, and vice versa, and different mold genera may produce the same mycotoxins.

Human health effects attributed to the inhalation of mycotoxins in workplaces include mucous membrane irritation, skin rash, nausea, immune system suppression, acute or chronic liver damage, acute or chronic central nervous system damage, endocrine effects, and cancer [9]. Furthermore, some nonspecific symptoms possibly related to mycotoxin production, such as cough, irritation of the eyes, skin, respiratory tract, joint aches, headache, and fatigue, have also been documented [10, 11]. Aflatoxin, trichothecenes, and ochratoxins are the most well-known mycotoxins found in the indoor environment [12, 13]. To date, several hundred mycotoxins have been discovered. Various methods, including high-performance liquid chromatography techniques such as gas chromatography-mass spectrometry (GC-MS) and liquid chromatography-mass spectrometry (LC-MS), are widely used for identifying and detecting mycotoxins. Liquid chromatography coupled with tandem mass spectrometry (LC-MS/MS) is vital and central for mycotoxin analysis. In contrast, the molecular polymerase chain reaction (PCR) approach and the enzyme-linked immunosorbent assay (ELISA) were commonly used in fungi identification [14]. The ability and sensitivity of multiple mycotoxin quantifications in various matrices such as food, feed, and biological samples have expanded significantly since 2010, attributed to the progress in LC-MS and the combination of appropriate sample extraction and cleanup procedures [15–17]. This approach is highly relevant as several mycotoxins tend to co-occur with others, regardless of the similarity in their chemical structure [18–20]. The most common mycotoxin extraction method applied for an indoor environment is liquid-liquid extraction (LLE) with a wide variety of solvents such as methanol [21–24], acetonitrile [25–27], and dichloromethane [28, 29]. In addition, the combination of the solvent mixture, e.g., methanol, dichloromethane, and ethyl acetate and chloroform and methanol, in different ratios has also been adopted [23, 30–32].

This selected study is a scoping review that aims to provide insight into the recent mycotoxin study and analysis in the building's environment using GC-MS and LC-MS/MS from the available literature. This review has also enabled us to identify the knowledge gaps and future potential research in this area. Validated and updated evidence from this review can assist professional bodies in the importance of this subject matter on human health and mitigation strategies.

## 2. Methodology

Scoping reviews provide an excellent approach to analyzing research findings on a particular topic. Scoping offers an overview of the literature, narrowing down the related study to match our targeted case, and finally summarising the main component, concepts, and the available data to give an insight into the gap that is available in the field [33, 34]. Hence, a scoping review was chosen to review research articles on the available analytical techniques that allow the optimum discovery and quantification of targeted mycotoxins relying on the mass and ion charge methods. The mass spectroscopy system offers sensitivity and specificity for challenging and matrix-complex samples. The selected topic accommodated the proposed research study in the Institute for Medical Research (IMR), Malaysia (NMRR-18-962-41809). For the scoping approach, we adopted Arksey and O'Malley's [35] method consisting of six phases to guide the selection process of the suitable literature available for our review purposes. We also considered updated guidelines published by the Preferred Reporting Items for Systematic Reviews and Meta-Analysis (PRISMA) 2020 statement [36].

### 2.1. Phase 1: Identifying the Research Question

Various methods have been adopted for determining mycotoxins, whether in the air, building materials, or dust, utilizing mass spectrometry or a more advanced tandem mass spectrometry method. As for our scope, we would like to answer “what are the recent methods of LC-MS/MS and GC-MS that are being used to determine the presence of mycotoxin in the targeted samples collected from indoor air in building environments?”

### 2.2. Phase 2: Identifying the Relevant Studies

During the search, the following criteria were set as guidance:

1. Articles were written in English only
2. Articles published from 2010 to 2020
3. Databases search from PubMed, Google Scholar, Cochrane, Scopus, Wiley, and ScienceDirect
4. Open-access research articles only, excluding review papers, book chapters, and conference papers
5. Keywords are indoor air, mycotoxin, LCMS/MS, and GCMS

### 2.3. Phase 3: Study Selection

**Figure 1:**
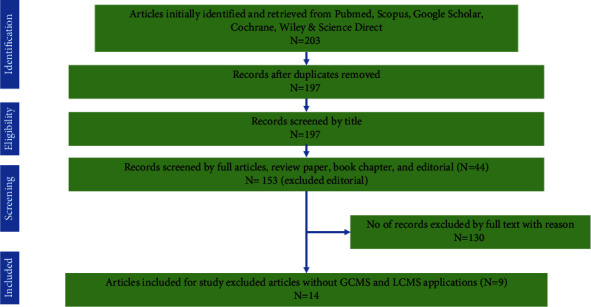
Flowchart of literature search strategy. PRISMA flowchart on literature selection based on inclusion/exclusion criteria to identify the occurrence of AR in the environment of dairy farms [37].

PRISMA guidelines were used in our study selection to assist as a guided protocol for the search. At the initial stage, all team members were assigned to a specific database to conduct a web search based on the selected keywords. The search was then transferred to the Microsoft Excel spreadsheet and assigned to team members to scrutinize the obtained papers based on our agreed inclusion criteria. Team members will review the journal papers' abstracts to determine their relevance to our review scope. Furthermore, the full articles will be retrieved, and the information will be transformed to highlight the findings to decide whether a particular paper will be included or excluded for scoping review purposes. When members were unsure about the acceptability of a particular paper during abstracts or full-text screening, a discussion session was conducted to finalize the decision. The flow of the process is shown in Figure 1.

### 2.4. Phase 4: Charting/Organizing the Data

The identified, sorted, and selected papers were charted into a table to organize the information systematically.  Table 1 shows how the information was tabulated to meet the research questions and scoping review purposes.

### 2.5. Phase 5: Collating, Summarising, and Reporting the Results

**Figure 2:**
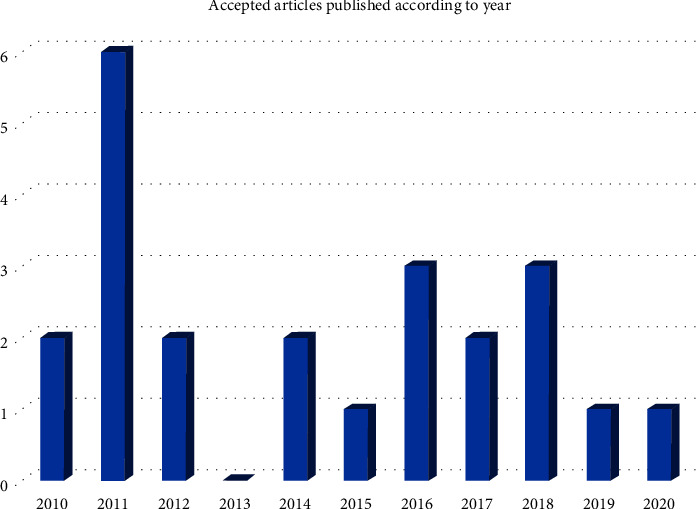
Accepted articles published according to year.

The main aim of a scoping review is to collect the available data, results from findings, and research output into more tangible information to be well-versed on the research that had taken place, the area that can be improvised, and finally give an overview of the research gap that can be tackled for the future research direction (Figure 2).

## 3. Mycotoxins in Buildings

This review was started with a concurrent investigation on mycotoxins in Peninsular Malaysia's healthcare institutions initiated by researchers from the Institute for Medical Research (IMR), Malaysia. A scoping review was published covering topics on fungal identification in hospital settings, with participants from many nations and locations [38]. The review was generally on fungus profiling in hospitals, with limited papers reviewed on mycotoxin distribution. The findings revealed that the most common fungal genera detected in such settings were *Aspergillus* sp., *Cladosporium* sp., *Penicillium* sp., and *Fusarium* sp. with the most identified *Aspergillus* sp. in hospital wards being *A. flavus*, *A. fumigatus*, and *A. niger*, when compared to several settings such as neonatal intensive care units (NICUs), labour rooms, laboratories, and others. The findings discovered that intensive care units (ICUs) and wards were home to various fungus species, primarily *Aspergillus* spp. (Sham et al., 2021). Additional information on analysis for secondary metabolites in indoor air in building environments reported in this current review believes that more studies in this area can be pursued to gain more knowledge and understanding to solve issues quickly and effectively.

In the past ten years (Jan 2010–Feb 2021), 14 published studies on analysis for secondary metabolites in indoor air in building environments were recorded. Five studies were published in 2016, followed by three earlier publications in 2011. Significant findings from analysis of secondary metabolites in indoor air and studies on building environments based on 14 selected articles are listed in Table 2. Most studies on these areas were conducted in European countries, with four studies in France [21–23, 32]. One of each study was conducted in Italy [39], Croatia [30], Poland [27], Denmark [31], and Germany [25], respectively. Two studies were conducted in Finland [25], but another was in collaboration with researchers from the Netherlands and Spain [29]. Three studies were reported from outside Europe, one from the USA [24], and the other two were conducted in Malaysia. Both studies collaborated with a researcher from Sweden [28, 40].

Indoor air samples and study locations were selected from water-damaged home buildings [21, 24, 25, 27], schools and kindergartens [28, 29, 31, 40], workplaces (farms and industries) [22, 23, 39], and few selected buildings and locations [30] (Vishwanat et al., 2011), but one study did not mention their location [32]. Regarding study scope and objective, most studies provided qualitative and quantitative descriptions of the microbial toxins in indoor air and their metabolites found in samples. Lanier et al. [22] conducted an in vitro study on a specific fungus to check on a specific mycotoxin produced. Jeżak et al. [27] determined the toxicogenic potential of fungus isolates from moldy surfaces. Other studies extended into mutagenic properties of bioaerosol samples [21, 22], health risk assessment [23, 28, 40], and cytotoxic potency [30]. The development of mycotoxin screening in airborne particulate matter and method performance using LC-MS was also described [39].

There are studies reported on mycobiota on building materials and bioaerosols collected from different selected locations [21, 22, 25, 30]. Pottier et al. [21] reported that nine out of twenty selected houses contained a fungus identified as *Serpula lacrymans*. Also found in the selected houses were ligninolytic strains like *Donkioporia expansa*, *Serpula himantioides*, and *Coniophora puteana*. Lanier et al. [22] identified 45 fungal species in the cattle shed where *Stachybotrys chartarum* was observed for the first time. Among the common fungal species identified were *Aspergillus fumigatus*, *Cladosporium cladosporioides*, *Penicillium chrysogenum*, *Stachybotrys chartarum*, *Ulocladium chartarum*, and *Aspergillus glaucus*. *Stachybotrys chartarum* was found to be the highest contribution of recurrent strain which was up to 41%. Fourteen fungus species (8 genera) and three yeast species (2 genera) were most frequently isolated on infected surfaces in residential rooms in Poland. They were identified as *Aspergillus versicolor*, *Cladosporium cladosporioides*, *Penicillium chrysogenum*, *Ulocladium chartarum*, and *Acremonium charticola*. Four identified genera were susceptible to humans (*Aspergillus* sp., *Penicillium* sp., *Cladosporium* sp., and *Phoma* sp.). These included two species capable of producing hazardous mycotoxins (*Aspergillus versicolor* and *Penicillium chrysogenum*) [27].

**Figure 3:**
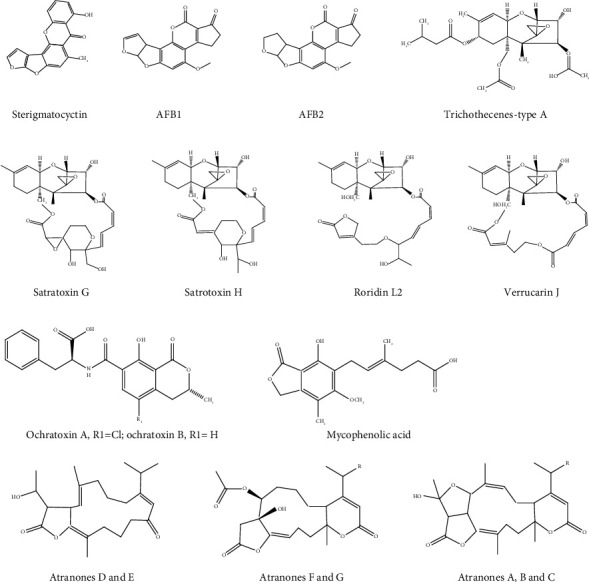
(a, b) Examples of some of the mycotoxin structures mentioned in text.

*Aspergillus* section *Versicolores* producing sterigmatocystin was found in an apartment's basements and grain mill, the first study over a year in Croatia. The dominant and highest sterigmatocystin-producing species identified using the calmodulin sequence were *A. jensenii* (1.192–133.63 *μ*g/mL), *A. creber*, and *A. griseoaurantiacus* (208.29 *μ*g/mL). The study also showed that the *Aspergillus* extracts producing positive-sterigmatocystin exert cytotoxicity towards A549 and THP-1 macrophage-like cells in low concentrations [30]. A fungal toxicogenic evaluation was conducted from a mold-infected exterior found in a residential room in the urban agglomeration in Poland without the influence of an environmental factor such as a flood-affected building or area. Mycological analysis showed *Aspergillus versicolor* and *Penicillium chrysogenum* producing sterigmatocystin (Figure 3(a)) and roquefortine C at a range of 2.1–235.9 *μ*g/g and 12.9–27.6 *μ*g/g, respectively, and the detection from air dust and scrapped material was below the limit of detection [27]. Indoor air quality greatly affects respiratory illnesses in which Norbäck et al. [40] found the prevalence of rhinitis and sick-building syndrome among students (*n* = 462; 14–16 years) from the tropical country of Malaysia. Total fungal DNA and Asp/Pen DNA were detected in all classrooms from Petri dishes and swab samples. In the Petri dish samples, 70% detection was obtained for *A. versicolor* DNA, 13% for *S. chartarum*, and 87% for *Streptomyces* DNA. Meanwhile, for swab samples, the detection was recorded at 56% for *A. versicolor* DNA, 3% for *S. chartarum* DNA, and 28% for *Streptomyces* DNA.

An interesting risk association existed between mycotoxin growing on wallpaper and the transfer to indoor air. A study by Aleksic et al. [32] showed mycotoxins growing on wallpaper followed by aerosolization from the infected surfaces produced macrocyclic trichothecenes (112.1 mg/m^2^) of satratoxins G and H, roridin L2 (RL2), and verrucarin J (VerJ); mycophenolic acid (1.8 mg/m^2^); and sterigmatocystin (27.8 mg/m^2^) as shown in Figures 3(a) and 3(b). The mycelium branching from fungal species and conidial morphology contributed to the aerosolization of particles from a substrate with the macrocyclic trichothecenes requiring the highest airspeed, and the total aerosolized toxic load was 5-fold more than others. *Stachybotrys chartarum* required the highest velocity of 5.9 m/s compared to *Aspergillus versicolor* and *Penicillium brevicompactum* to transfer the contaminated substrates. *Stachybotrys chartarum* biomarkers were identified in pure fungal cultures and cotton-tipped swab extracts collected from kindergarten in Greater Copenhagen. The identification revealed 12 *Stachybotrys* metabolites with atranones and macrocyclic trichothecenes (Figure 3(b)) on the gypsum wallboard. Došen et al. [31] also reported that it was the first time the same mycotoxins were found on the contaminated gypsum wallboard and settled dust. Four mycotoxins were targeted at water recycling and recovery facilities in France. Ninety-four air samples revealed quantifiable aflatoxin B1 and sterigmatocystin, while gliotoxin and ochratoxin (Figure 3(b)) were not found in any samples. Mycotoxin exposure was reported to be insignificant and did not give any concerning threat to the workers in a study conducted at waste management facilities [23]. A comparative study using settled floor dust collected from waste management facilities in Germany and residential houses in Finland showed a wider range of metabolites in concentrations of 0.04–49, 1444.0 *μ*g/kg (Vishwanath et al., 2011).

### 3.1. Sample Collection and Processing

The sample collection and extraction procedures for mycotoxin analysis are summarised in Table 3. Mold and mycotoxins were sampled using a variety of techniques. Building material and dust samples from damaged buildings were collected using a vacuum cleaner [25] to detect multiple microbial toxins from indoor samples and naturally infested materials. Two papers described airborne dust analysis by sampling samples using cotton swabs and Petri dishes. This is performed in a classroom where they are interested in the associations of respiratory symptoms with the levels of selected fungal DNA, furry pet allergens, and mycotoxins in schools [28, 40]. A vacuum cleaner was used to collect settled dust floored from houses inhabited by small groups of people, generally less than 5 (Vishwanath et al., 2011). A foam swab wetted with methanol and swiped across the sampling area was performed for settled dust and moldy spot swab surfaces at different sites in school buildings [29]. Scraping on moldy surfaces and airborne dust samples inside residential rooms were collected using the “aspirator and head with filter” sets. The set consisted of a GilAir-5 (Sensidyne, USA) aspirator, an elastic hose, and an open-measuring head (Two-Met, Poland), with 37 mm diameter and 0.7 *μ*m pore diameter of the GF/F glass fiber filter (Whatman, UK) reported by [27]. Pottier et al. used two methods to collect fungal aerosols in a damaged house: a sterile polytetrafluoroethylene (PTFE) filter with a 0.2 *μ*m pore size attached to a calibrated vacuum pump and a sterile liquid with a cyclonic air sampler Coriolis® (Bertin Technologies, France). PM4 and PM10 samplings were performed for indoor/outdoor environments [39]. Another study on ambient air PM10 sampling was reported where bioaerosol from a cattle shed monitored revealed the presence of mycotoxins without concentration data due to below quantification unit. Airborne fungi were collected using a MAS-100 Eco air sampler (Merck, Darmstadt, Germany) with 400 holes (hole to agar impactor) and dichloran 18% glycerol agar (DG18) plates [30]. Air sampling was carried out by collecting dust with a CIP 10 sampler. The sampler uses the rotative cup technique with rotation, maintaining a flow rate of 10 L·min^−1^. The sampler cup had a porous polyurethane foam filter (PUF). After sampling, the rotating cup containing the PUF was removed from the sampler, closed by the cover, and stored at 4^○^C before analysis [23].

Floor dust mycotoxins were reported by [24] where they used a vacuum attached to a polyethylene filter sock (Midwest Filtration Company, Fairfield, OH, USA) and a precleaned crevice tool on a L'il Hummer™ backpack vacuum sampler (100 ft3/min, 1.5 horsepower; ProTeam Inc., Boise, ID, USA). An Andersen multistage impactor (Tish 180 Environmental, OH, USA) was used for capturing particles according to 6 ranges of size and 181 aerodynamic characteristics. Each impactor stage had a fiberglass disk to collect particles [32]. Settled dust samples were collected from all available surfaces (shelves, tables, fridges, and tops of the hanging lamps) and other places (excluding the floor) that were regularly cleaned. Each sample was taken from an approximate surface area of 45 × 45 cm using a clean precision Kimwipes® Lite wipe (Kimberly-Clark, GA, USA). Pure agar cultures were extracted using a microscale method modified for *Stachybotrys* metabolites. Three agar plugs (6 mm ID) were cut from a 15-day-old colony from each agar medium (potato dextrose agar (PDA) or malt-extract agar (MEA)) and placed in a 2 mL screw-top vial. Extracts from pure fungal cultures and cotton tip swabs from infected gypsum wallboards were further processed in laboratory before analysis for detection of mycotoxin metabolites by injection directly to an ultrahigh performance liquid chromatography diode array detector quadrapole time-of-flight mass spectrometry method (UHPLC-DAD_QTOF/MS) [31].

Depending on the type of samples collected for sampling, it is important to note that sampling techniques may differ depending on the specific objectives, environment, and suspected mycotoxin contamination sources. The vacuum cleaner can cover a large sampling area and larger sample material. At the same time, cotton swabs and Petri dishes allow for targeted sampling of specific areas where mold growth is visible. The GilAir-5 aspirator is a portable air sampling pump commonly used to collect and analyze various contaminants, including gases, vapors, and aerosols.

The method of collection and the type of samples obtained are the main differences between a sterile PTFE filter with a 0.2 m pore size attached to a calibrated vacuum pump and a sterile liquid with a cyclonic air sampler Coriolis®. A sterile PTFE filter with a pore size of 0.2 m connected to a calibrated vacuum pump is commonly used to collect particulate matter such as dust, pollen, or other solid particles in the air. The filter serves as a barrier, trapping particles while allowing air to pass through. The filter can be removed and analyzed after sampling to determine the types and quantities of particles present. On the other hand, a sterile liquid with a cyclonic air sampler Coriolis® collects microorganisms, such as bacteria and fungi, from the air. The cyclonic action within the sampler separates and concentrates the airborne microorganisms onto a sterile liquid substrate. This liquid is then used for laboratory analysis to identify and quantify the microbial contamination present in the air.

The main difference between PM10 and PM4 air filters lies in the size range of particles they are designed to capture. A PM10 air filter is specifically engineered to capture particles 10 *μ*m or smaller in diameter, including dust, pollen, mold spores, and larger airborne particles. By targeting this size range, the PM10 filter helps monitor and assess air quality, as these larger particles can potentially impact respiratory health and indoor or outdoor air pollution levels. On the other hand, a PM4 air filter is designed to capture particles that are 4 *μ*m or smaller in diameter, including finer particles, such as combustion byproducts, soot, fine dust, and certain allergens. By focusing on this smaller particle size, the PM4 filter provides more detailed information about fine particulate matter, which is known to have potential health implications, especially when inhaled.

The MAS-100 Eco air sampler is an advanced air quality monitoring and analysis device. It is specifically designed to sample and measure microbial contamination in the air, including bacteria, fungi, and other microorganisms. The MAS-100 Eco air sampler utilizes a high-performance filtration system to capture and collect these microorganisms, permitting further analysis and identification in laboratories. A CIP 10 sampler is an individual sampler that traps respirable particles. The physical collection efficiency of CIP 10 equipped with the inhalable fraction selector is estimated to be 50% for particles with an aerodynamic diameter of 1.8 mm and more than 95% for particles with an aerodynamic diameter greater than 2.8 mm.

### 3.2. Instrumentation Analysis

After sampling, mycotoxins require sample preparation using appropriate analytical instruments. Sample preparation demands using a suitable solvent to extract toxins from the matrix, a cleanup procedure to remove interferences from the matrix, and, if necessary, sample preconcentration before analysis. Selecting an appropriate solvent for mycotoxin extraction depends on the toxin's structure. The most common method is liquid-liquid extraction (LLE), as shown in Table 3. Different types of solvent were used for the extraction of mycotoxins, such as methanol [21–23] and acetonitrile [25–27], and dichloromethane [28, 29]. Alternatively, a combination of a solvent mixture such as methanol, dichloromethane, and ethyl acetate [30, 31] and chloroform and methanol (2 : 1) [23, 32] was performed to extract different metabolites from samples which are compatible with the solvent. One paper reported on the two-stage extraction (methanol followed by hexane) procedure [40]. One paper conducted sampling on headspace-solid-phase microextraction (SPME), which employs a fiber coated with an extracting phase, which can be a liquid (polymer) or a solid (sorbent), to extract various analytes (volatile and nonvolatile) from various media (Vishwanath et al., 2011). Another paper described an accelerated solvent extractor (ASE) step followed by solid-phase extraction (SPE), ultimately enhancing the purification of analytes, making it possible to eliminate, reduce, and suppress signals from interference [39].

Airborne and bioaerosol samples were analyzed for instrumentation analysis using two HPLC-MS/MS protocols to cover many mycotoxins. Positive and negative ion modes were chosen to obtain a good signal from mycotoxins [21, 22]. One study was reported on the ultraperformance liquid chromatography (UPLC) system connected to Xevo Triple Quadrupole to determine mycophenolic acid, sterigmatocystin, and macrocyclic trichothecenes [32], UPLC-Orbitrap [23], and UHPLC-QTOF [31]. One paper reported mycotoxins in dust analyzed by gas chromatography-MS/MS [24], and one paper described mycotoxins indoor/outdoor airborne particulate matter using LC-MS/MS both in positive and negative ion modes to achieve efficient ionization for the known analytes [39]. Combination analysis using GC-MS/MS and LC-MS/MS was competent to cover volatile and nonvolatile compounds. These instruments analyzed airborne dust, fungi, and moldy surface samples [25, 28, 29, 40]. Interestingly, one paper reported using headspace GC-MS, and detection and quantification were performed on QTRAP LC-MS/MS (Vishwanath et al., 2011). Two papers described the application of HPLC to detect mycotoxins. Concentrations were calculated based on peak areas of the analyte compared to calibrated standards [27, 30].

Table 4 shows that most of the mycotoxins are analyzed using LC-MS/MS compared to the GC-MS method. Twelve publications reported mycotoxin analysis by LC and differentiated by HPLC, HPLC-MS/MS, LC-MS/MS, HPLC/UV-VIS, UPLC, and UHPLC. In general, mycotoxins such as aflatoxins (AFB1, B2, G1, G2, and M1), gliotoxin, mycophenolic acid, ochratoxin, alternariol, zearalenone, sterigmatocystin, patulin, citrinin, fumagillin, and trichothecenes (neosolaniol, T-2 toxin, HT-2 toxin, nivalenol, satratoxin, and roridin) are frequently quantified using liquid chromatography with the detector of a mass spectrometer. All the data acquisition on LC-MS/MS in mycotoxin experiments was performed in the positive or negative ESI (electrospray ionization) mode, using multiple reaction monitoring (MRM) scans.

Although some of the mycotoxins like sterigmatocystin are analyzed using detectors other than MS, e.g., UV-VIS [30], one of the advantages of MS over the other detectors is that it is easier to distinguish coeluting compounds using extracted ion chromatograms. LC-MS/MS offers a sensitive, efficient, and multianalyte analysis which is of great importance, especially on mycotoxin determination in various matrices, including indoor environmental samples, for example, ambient air, settled dust, and moldy surface. Attempts to identify these toxins in dust particularly are challenging as it correlates to the amounts present in the sample. Many fungal metabolites possess the same elemental composition and coeluate at the same retention time. Thus, a specific and sensitive instrument is required to distinguish similar compounds which are normally difficult to separate chromatographically. Indeed, several LC-MS/MS multimethods (≥2 mycotoxins) have already been developed for indoor environmental samples [28] (Vishwanath et al., 2011). Developed methods were reported to produce good recovery ranging from 42 to 101.10% with CV around 10% and *R*^2^ of 0.994–0.999 (Table 4).

### 3.3. Method Validation

GC-MS and LC-MS/MS are widely used techniques for mycotoxin analysis in various environmental samples, including building materials, due to their high sensitivity, selectivity, and ability to analyze complex matrices. These techniques have successfully identified and quantified a broad range of mycotoxins, even at low levels, and can differentiate between mycotoxin isomers and closely related compounds. However, the reliability and accuracy of these techniques depend on proper method development, validation, and quality control measures. The lack of such data in studies significantly impacts the interpretation and outcomes. Without proper validation and quality control, findings may be influenced by matrix interferences, extraction efficiency, instrument variability, and method biases. Thorough evaluation and reporting of method performance parameters, such as limit of detection (LOD), limit of quantitation (LOQ), linearity, accuracy, precision, and selectivity, are essential. Quality control measures, including calibration standards, matrix-matched standards, and internal standards, are crucial to ensure accuracy and reliability. In addition, using appropriate quality control samples, such as certified reference materials, helps assess measurement uncertainty and ensures comparability across studies.

In mycotoxin analysis, it is crucial to mitigate matrix effects to ensure accurate and reliable results. Matrix effects occur when the sample matrix interferes with the ionization and detection of analytes, resulting in signal suppression or enhancement. To ensure optimal performance, assessing the practices employed to mitigate matrix effects (e.g., matrix-matched calibration, internal standards, and different sample preparation techniques) is essential.

To obtain an accurate measurement, matrix-matched standards to reduce matrix effects [24], stable isotope-labelled internal standards such as the 13C standard [21], and efficient sample cleanup [39] are normally performed. The detection and quantification of an analyte are significantly influenced by matrix effects associated with heterogeneous components in environmental samples [44]. Coextracted matrix components may cause interference with active sites in the GC inlet liner and the column and produce differential analyte signals between the matrix-containing sample extract and the matrix-free standard extract [45–47]. Efficient sample preparation, for example, solid-phase extraction (SPE) or LLE, is essential and has been found to reduce matrix effects potentially [48]. The effectiveness of these techniques in reducing matrix effects can vary depending on the sample matrix and mycotoxin of interest. This step is crucial, and optimization is needed to minimize sample loss. An internal standard (IS) is frequently used to improve the precision of quantitative analysis in which it compensates for matrix effects or sample loss during preparative procedures. In other words, it monitors and corrects any variations during sample preparation, extraction efficiency, and instrument response. Therefore, the selected IS should be similar to the target analytes regarding ionization properties or chemical structures to ensure it always reacts the same way as the analytes of interest, especially with a matrix [49]. An IS labelled with (13C) or (15N) was commonly employed for each group of mycotoxins, for instance, Fumonisin B1-13C34 and Deoxynivalenol-13C15 [21, 22, 39]. However, a nonlabelled IS, such as reserpine, has also been used as an internal standard [40]. According to Saito et al. [24], the accuracy of the results is largely dependent on the matrix effects, the appropriateness of IS, or the combination of them. On the other hand, only five publications reported analyses have been performed using GC-MS with the negative chemical ionization (CI) mode. Trichodermol and verrucarol, which are in the group of trichothecenes, are the most common mycotoxins tested by GC-MS [24, 28, 29, 40] (Vishwanath et al., 2011).

## 4. Conclusions

In conclusion, various techniques are available for analyzing and detecting multiple mycotoxins using LC-MS/MS and GC-MS methods. Mold and mycotoxin analysis has evolved in sampling techniques, processing, preconcentration, and instrumentation over the past years. Technological advances are beginning to overcome many challenges posed by the complexity of detecting multiple mycotoxins. Mass spectrometry advancements such as ionization modes, sensitivity, and acquisition speed have increased throughput, the number of mycotoxins that can be simultaneously screened, and the discovery of novel compounds of mycotoxins. Modern technologies, such as hyphenated liquid or gas mass spectrometry, have enabled these analytical methods to be developed and validated for mycotoxin analysis. However, due to the variety of chemical structures, using a single method for mycotoxin analysis is impossible. Routine analysis faces significant challenges due to the demand for rapid, simultaneous, and accurate determination of multiple mycotoxins. Future efforts would concentrate on rapid assays and tools that measure a broader range of mycotoxins in a single matrix and lower detection limits. Highly sophisticated multianalyte methods based on liquid chromatography coupled with multiple-stage mass spectrometry have been developed to identify and determine multiple mycotoxins. This new era of various screening mycotoxin and detection technologies will benefit future research.

## 5. Future Perspectives

It is anticipated analytical techniques and technologies for mycotoxin detection are likely to advance. This could include creating more sensitive and specific methods, such as advanced chromatographic techniques or rapid screening methods based on biosensors or nanomaterials. These advances will allow for faster and more accurate detection of mycotoxins in a variety of samples.

Moreover, there will be a greater emphasis on developing portable and field-deployable mycotoxin analysis devices. This will enable on-site testing and real-time monitoring, which is especially important in monitoring building environments where rapid decisions are required to prevent mycotoxin contamination.

## Figures and Tables

**Table 1:** Search strategy for identification of studies.

No.	Keywords
1	Mycotoxin
2	Indoor air
3	LCMS/MS
4	GCMS
5	Combination of 1 AND 2 AND 3
6	Combination of 1 AND 2 AND 4
7	Combination of 1 AND 2 AND 3 AND 4

**Table 2:** Highlights on findings of analysis for secondary metabolites in indoor air and studies of building environments based on 14 selected articles.

No.	Country	Samples and study locations	Study aims	Highlights on important findings	Reference
1	France	Fungal aerosols were collected from homes damaged by wood-rotting fungi in Lower Normandy, France. Damaged homes were described as having high moisture content, lack of ventilation, and improper renovation	The fungal contamination in homes harmed by wood-decaying and airborne molds*, Serpula lacrymans*, was described in this study. Mycotoxins in the air were measured using HPLC-MS/MS, and the Ames test was used to determine the mutagenicity of fungal aerosols. Fungal species growing on building materials were studied using cultural and molecular methods, mycotoxins in the air were measured using HPLC-MS/MS, and the Ames test was used to determine the mutagenicity of fungal aerosols	Results showed that *Serpula lacrymans* was detected in the air for one-third of homes with sometimes the co-occurrence of other ligninolytic basidiomycetes species like *Donkioporia expansa*. Various molds in the air (117 species) and on materials (103 species) were identified including a recurrent species like *Aspergillus versicolor* and *Penicillium fellutanum*. Airborne culturable fungal levels were measured up to 5.8 × 10^5^ CFU/m^3^. Alternariol and/or ochratoxin A mycotoxins were observed in 4 homes, but no mutagenic activity was found	[21]

2	Italy	Atmospheric particulate matter was sampled in wet indoor environment, workplaces with previous flood and fungal spores detected, and outdoor environments. No specific sampling location was mentioned	Screening of mycotoxins (deoxynivalenol, aflatoxin B1, ochratoxin A, T-2 toxin, zearalenone, and sterigmatocystin) in indoor and outdoor environments, airborne particulate matter was developed, and method performance data were presented	Single-step extraction using optimal solvent mixture (acetonitrile: water at 90 : 10) utilizing the accelerated solvent extraction method and purified using solid-phase extraction via Strata C18-M cartridges. Good linearity was obtained for each mycotoxin at different concentrations by correlation coefficients ranging from 0.994 to 0.999. The apparent recoveries from HPLC/ESI-MS/MS analysis in the MRM mode were >70% with a 10% CV, and the optimized process allowed simultaneous separation of analyte in less than 10 minutes	[39]

3	France	Bioaerosol samples were collected from cattle sheds located in Normandy	To analyze fungal contamination of bioaerosols collected in a dairy shed during two periods of 10 days each, investigate the toxigenic capacities of *Aspergillus fumigatus* and *Aspergillus flavus* group isolates, and use the Ames test to determine the mutagenic properties of bioaerosol samples	*Aspergillus flavus, Aspergillus fumigatus, Penicillium chrysogenum, Stachybotrys chartarum*, and the allergenic species *Ulocladium chartarum* and *Cladosporium cladosporioides* were the most common in the air. Between the two study periods, the median CFU/m^3^ ranged from 6.48 to 76.4, which exhibited significant differences between species. During straw processing, there was a peak of fungal contamination. In vitro *A. flavus* isolates do not produce aflatoxin, while *A. fumigatus* isolates do produce gliotoxin, verruculogen, and fumagillin. Aflatoxin B1 (0.09 ng/filter) and B2 (0.07 ng/filter) were found in bioaerosols at concentrations below LOQ. Results showed that farmers may be exposed to *Stachybotrys chartarum* during ordinary barn work	[22]

4	Finland	Airborne dust samples were collected from surfaces above floor level of single-family homes across Finland	In this work, sample materials from severely moisture-damaged dwellings were subjected to a multianalyte tandem mass spectrometry-based technique. The goal is to provide a qualitative and quantitative description of the various microbial metabolites found in the samples	Indoor samples for at least one of the 186 analytes analyzed yielded 69 positive results, with up to 33 different microbial metabolites discovered. Hazardous bacterial metabolites were discovered in indoor samples, along with their co-occurrence with mycotoxins. The bacterial chemicals including monactin, nonactin, staurosporine, and valinomycin were found in moist building materials, whereas chloramphenicol was found in high concentrations in house dusts, including settled airborne dust. *Streptomyces* spp., a species of microbes that is considered a moisture damage indicator in indoor environments, produces these bacterial metabolites, which are highly bioactive chemicals	[25]

5	Malaysia	Airborne dust was collected from randomly selected secondary schools and classrooms in Johor Bahru, Malaysia. A questionnaire with standardized questions was used for health assessment in 15 randomly selected pupils from each class	The researchers wanted to look for specific fungal DNA, hairy pet allergies, and mycotoxins in dust samples from Malaysian schools and see if there were any correlations with kids' respiratory health	Results indicated that fungus DNA and cat allergens were abundant, and students had a high rate of doctor-diagnosed asthma and respiratory problems. The finding of positive associations with specific DNA from the fungal species *A. versicolor* and the bacteria *Streptomyces* spp., despite the lack of a link between total fungal DNA and respiratory health, highlights the importance of analyzing specific microbes when studying respiratory health effects. The use of qPCR to analyze fungal and bacterial DNA collected using the Petri dish sample method appears to be a promising tool for monitoring indoor mold exposure because it can detect both general and DNA sequences regardless of whether organisms are alive or dead	[28]

6	Finland The Netherlands Spain	The settled dust swab and moldy spot surface swab samples were collected from different locations in school buildings in Spain, the Netherlands, and Finland	The study measured both buildings with and without moisture damage and/or dampness observations. More than 180 analytes were targeted in settled dust and surface swab samples using the LC/MS-based methodology	The results showed that 42%, 58%, and 44% of all samples collected in Spanish, Dutch, and Finnish schools, respectively, were positive for at least one of the metabolites analyzed. The frequency of microbial secondary metabolite determination (except for emodin, certain enniatins, and physcion) was low and ranges from 10% and below of positive samples. Thirty different fungal and bacterial secondary metabolites were found in samples. Some differences in the metabolite profiles were observed between countries and between index and reference school buildings. A major finding was that settled dust from contaminated schools contained additional higher levels of microbial secondary metabolites than dust samples from noncontaminated schools	[29]

7	Croatia	Indoor air samples of selected apartments, basements, and grain mills were collected in Croatia	The distribution and species diversity of sterigmatocystin (STC)-producing Aspergilli from the section Versicolores in the indoor air of apartments (APs), basements (BS), and grain mills (GMs) in Croatia, as well as their cytotoxic potency, are presented in this study	Total airborne fungal species detected in sampling locations: 0.7–20% in the AP, 11–55% in the BS, and 0–2% in the GM. Dominant species were *A. jensenii* and *A. creber*, followed by *A. protuberus, A. venenatus, A. tennesseensis, A. amoenus, A. griseoaurantiacus,* and 3 undescribed species. Species that produced STC: *A. griseoaurantiacus* (208.29 *μ*g/mL), *A. jensenii* (1.192–133.63 *μ*g/mL), and *A. protuberus* and *A. tennesseensis* (0.117–2.749 *μ*g/mL). Lower species diversity was obtained in the GM with relatively high STC levels (0.06–2.35 *μ*g/g) in 52% samples. STC cannot be fully attributed to Aspergilli (Versicolores). Human lung A549 cells and THP-1 macrophage-like cells were cytotoxic to STC and most STC-producing aspergilli at relatively low concentrations, indicating that humans are at high risk during chronic exposure	[30]

8	Poland	Urban agglomeration in central Poland: Scrapping and airborne dust samples were collected from residences selected from flats by a simple random selection method. Selection was based on the existence of mycelium on solid surfaces in the flats	The major goal was to determine the toxinogenic potential of fungus isolated from moldy surfaces in residential rooms in an urban agglomeration, remote from flooded areas in a mild temperate zone	This study confirmed on capability of producing sterigmatocystin and roquefortine C by growing *Aspergillus versicolor* and *Penicillium chrysogenum,* respectively, in mixture of fungi from scraping and pure cultures in laboratory conditions. Results shows that sterigmatocystin was produced by 8/13 isolated strains of *Aspergillus versicolor* (2.1–235.9 *μ*g/g), while 4/10 of isolated strains of *Penicillium chrysogenum* produced roquefortine (12.9–27.6 *μ*g/g). Sterigmatocystin was determined (3.1–1683.2 *μ*g/g) in 11/13 scraping samples positive with *Aspergillus versicolor*, while *Penicillium chrysogenum* was determined in 3/10 of samples with roquefortine C (0.9–618.9 *μ*g/g). Mycotoxins in all air dust and scraping samples were detected below LOD, indicating minor exposure to residents in moldy flats of urban agglomeration situated far from flooded territories	[27]

9	Malaysia	Settled airborne dust was collected from randomly selected 8 primary schools in Johor Bahru, Malaysia. 462 students from four classes of grade two were selected to participate in the questionnaire survey	To see if there was a link between rhinitis and other types of weekly SBS symptoms among junior high school students in Johor Bahru, Malaysia, and levels of cat allergen (Fel d 1), two mycotoxins (verrucarol and sterigmatocystin), and five fungal DNA sequences in the classrooms	Indoor CO_2_ levels were 492 ppm (range 380–690 ppm) in all classrooms with open windows. Fungal DNA and cat allergens were common in the studied Malaysian schools, and there were a high prevalence of rhinitis and SBS symptoms among the students, especially headache (20.6%) and tiredness (22.1%). Total fungal DNA in swab samples was significantly associated with rhinitis, ocular symptoms, and tiredness. Positive associations were shown between *Aspergillus versicolor* DNA in Petri dish samples, ocular symptoms, and tiredness. The level of the mycotoxin verrucarol in swab samples was positively associated with tiredness. *Streptomyces* DNA in swab samples and Petri dish samples were negatively associated with tiredness. Mycotoxins may be ubiquitous in tropical indoor environments	[40]

10	France	Aerosolization of mycotoxins after growth of toxinogenic fungi on wallpaper. No specific sampling location was mentioned	This study investigated mycotoxin production by *Penicillium brevicompactum*, *Aspergillus versicolor*, and *Stachybotrys chartarum* during their growth on wallpaper and the possible subsequent aerosolization of produced mycotoxins from contaminated substrates	Findings showed that mycophenolic acid, sterigmatocystin, and macrocyclic trichothecenes (sum of 4 major compounds) could be produced at levels of 1.8, 112.1, and 27.8 mg/m^2^, respectively, on wallpaper, and part of the produced toxins could be aerosolized from substrate. The propensity to aerosolization differed according to the fungal species. Particles were aerosolized from wallpaper contaminated with *P. brevicompactum* when air velocity of just 0.3 m/s was applied, where *S. chartarum* required 5.9 m/s, while *A versicolor* was intermediate at 2 m/s. Quantification of the toxic content revealed the association with particles of size equal to or higher than 3 *μ*m, which may correspond to spores. Some macrocyclic trichothecenes (especially satratoxin H and verrucarin J) can also be found on smaller particles	[32]

11	Denmark	A water-damaged daycare in the greater Copenhagen area provided fungal biomass and settled dust samples	For known and provisionally identified chemicals, an ultrahigh performance liquid chromatography-tandem mass spectrometry (UHPLC-MS/MS) approach was established using UHPLC-quadruple time-of-flight (QTOF) screening of fungal culture extracts, wall scrapings, and reference standards	This method discovered 12 *Stachybotrys* metabolites, which were measured using either real standards or standards that were similar to authentic standards. The two recognized chemotypes, S and A, coexisted in samples taken from *S. chartarum*-infected walls. A link between mycotoxin concentrations found on contaminated surfaces and in settled dust was established. Results from one dust sample collected from water-damaged room contained 10 pg/cm^2^ macrocyclic trichothecenes (roridin E). Primarily, more than one spirocyclic drimane was detected in dust where up to 600 pg/cm^2^ was detected in the water-damaged room and 340 pg/cm^2^ was detected in the adjacent room. They could be attractive candidates for exposure indicators because of their widespread dispersion in detectable concentrations in dust	[31]

12	France	Air samples were taken from several locations in France, including mechanical-biological treatment plants, large-scale industrial composting operations, and a material recovery facility	The purpose of this research was to find out how much airborne aflatoxin B1, ochratoxin A, gliotoxin, and sterigmatocystin were present in waste recycling and recovery facilities, as well as the health risks that came with it. Using ultrahigh resolution mass spectrometry and ultraperformance liquid chromatography, targeted mycotoxins were measured in 94 air samples collected in five locations	The results revealed that just 11% of the samples could be quantified. Mechanical separation areas in mechanical-biological treatment facilities and the material recovery facility were used to measure aflatoxin B1 and sterigmatocystin. All the exposure levels were less than 1 ng·m^−3^. This is the first time sterigmatocystin exposure in waste management facilities has been quantified. In any of the air samples, ochratoxin A and gliotoxin were not detected. Approaches to assessing health risks did not reveal a serious hazard to employees' health. Data did not support the requirement for specific preventative actions against the measured biological agent in the air	[23]

13	Germany	Settled floor dust samples were collected from different sites in Germany: waste management units dealing with municipal waste or paper recycling and severe moisture damage/dampness problems in houses occupied by less than 5 people	The determination of many biological and anthropogenic contaminants in settled floor dust using LC-MS/MS and GC-MS technologies is described in this paper (SFD). The presence of both microbial and nonmicrobial volatile organic molecules was determined using the GC-MS technique. The goal of the targeted LC-MS/MS study is to identify species-specific secondary metabolites	In the SFD matrix, 30 of the 71 discovered volatile organic compounds (VOCs) are new to the database. The results showed that using “AMDIS and SpectConnect” together was helpful in evaluating and identifying prime volatile contaminants in complex environmental samples. Nonanal was found as a possible MVOC marker using principal component analysis (PCA) of peak areas of 18 microbial volatile organic compounds (MVOCs). When considering their possible other origins from paints and cosmetics, respectively, toluene and 1-tetradecanolshowed discriminative influence but are not considered MVOC indicators	[25]

**Table 3:** Various procedures for mycotoxin studies in indoor air.

No.	Country	Sample collection	Type of extraction	Protocol/modification	Advantages/disadvantages	Reference
1	France	Fungal aerosols were collected for mycotoxin quantification by using a sterile PTFE filter of 0.2 *μ*m pore size mounted in 47 mm diameter filter holders and connected to personal air pumps calibrated to draw 2 L/min for 3 h. Sampling point was selected at room with visible damage and room without apparent damage	The mycotoxin from the fungal aerosol was extracted from the PTFE filters using 10 mL of methanol/water/formic acid (80/20/0.1), which was then maintained in an ultrasonic bath for 3 minutes before being agitated in a multitube vortex. Second extraction was performed with 10 mL of 50/50 dichloromethane/ethyl acetate	The extracts were combined and evaporated to dryness under nitrogen using a Buchi evaporator. The final residue was diluted in 0.5 mL of acetonitrile/water (10/90) and filtered through Millex-HV 0.45 m before being injected into the HPLC-MS/MS system	The study found that a multianalyte tandem mass spectrometry-based technology could be used to look for mycotoxins in fungal aerosols, allowing occupants' exposure to indoor mycotoxins to be assessed. Future research is needed to better understand the amounts of mycotoxins in indoor aerosols over lengthy periods of time	[21]
2	Italy	Indoors, a low-volume universal XR pump, SKC deluxe 224-PCEX8, fitted with an aluminium cyclone, was utilized at a flow rate of 2.5 L·min1 (to collect PM4), while an outdoor dual-channel sampler (HYDRA dual sampler) was employed at a flow rate of 2.3 m^3^/h (38.3 L/min) to collect PM10. Private interior environment, public indoor environment, indoor workplace, and outdoor environment sampling points were chosen	ASE extraction and followed by SPE purification	Sampled filters, blank filters, and spiked filters were extracted by ASE using two cycles of a combination of H_2_O: ACN (10/90) at 100°C and 1500 psi. The extracted volume (about 20 mL) was evaporated to dryness and redissolved in 1 mL water before loading into an SPE C18-M Strata cartridge that had previously been conditioned with 4 mL MeOH and rinsed with 4 mL water. After washing with pure water, compounds loaded and retained in the cartridge were eluted with 9 mL of MeOH/H_2_O 90/10 and 4.5 mL of pure ACN (4 mL). The analytes were swiftly eluted from the SPE cartridge at a steady flow rate using a vacuum manifold. Before the HPLC-MS-MS analysis, the extracts were evaporated under nitrogen stream. Optimal ASE extraction conditions: ACN/H_2_O (90/10) solvent mixture with two static extraction cycles (heat-up time 5 min; static time 5; flush volume 60%; purge time 300 s; pressure 1500 psi; T 100°C). The target analytes recovered the best with the Strata C18-M cartridge	Sample cleanup and SPE purification step reduced ion suppression significantly and/or eliminate interferences before HPLC-MS-MS analysis	[39]
3	US	21 dust floor samples from two water-damaged buildings were collected using floor vacuum. Each floor was vacuum covered for a 2 m^2^ area for 5 minutes. Samples were homogenized after removing hair, fluff, and larger objects	Extraction with methanol followed by derivatization	HP-5 ms fused-silica capillary column (30 m i.d., 0.25 mm i.d.). The sample injection volume is 1 l. The oven was set to a 70°C beginning temperature, which was then ramped up to 280°C at a rate of 20°C/min. The verrucarol derivative precursor ion was m/z 638, which gave a target product ion of m/z 302 for quantification and two ions of m/z 262 and 213 for qualification (retention time: 9.8 min). Internal benchmark (ISTD, 1, 12-dodecanediol)	Matrix-matched standard curves could be effective for obtaining accurate MCT readings in dust. In the dust extracts, ISTD showed significantly larger matrix effects. None of the 21 dust samples obtained from water-damaged buildings could be detected using standard calibration curves with ISTD modification	[24]
4	France	Ambient air sampling was conducted 24 hours per day in the feeding corridor cattle shed of dairy farm, using a high-volume sampler with PM10 head and 150 mm microfiber quartz filters. Sampling point was selected at feeding corridor	Samples were extracted twice with 30 mL of methanol acidified with acetic acid (0.5%) from the quartz microfiber filter. The solutions were kept in an ultrasonic bath for 3 min and then shaken for 10 min in a multitube vortexer. After evaporation to dryness, the final residue was dissolved in 0.5 mL of a mixture of acetonitrile/water (10/90)	Two approaches were created for the analysis. At 60°C column temperature, the first approach used a Zorbax Eclipse Plus column with a rapid resolution HD-C18 column (1.8 m, 50 2.1 mm; Agilent Technologies). Deoxynivalenol-13C15 was used as an internal standard. The injection volume was 20 *μ*L. Mixture of methanol (solvent A) and water (solvent B) as the mobile phase, linear gradient with 10%–100% solvent A for 10 min, and stay at 100% for 1 min, flow rate of 0.4 mL/min (positive and negative mode). In second approach, Zorbax SB, rapid resolution HT-C18 column (1.8 *μ*m, 50 × 2.1 mm; Agilent Technologies) at 60°C column temperature. Fumonisin B1-13C34 was used as the internal standard. The injection volume was 10 *μ*L. Mixture of acetonitrile (solvent A) and water added formic acid 1% (solvent B) as mobile phase, linear gradient with 10%–100% solvent A for 10 min, and stay at 100% for 1 min, flow rate of 0.4 mL/min (positive mode)	NIL	[22]
5	Finland and Sweden	Settled airborne dust samples were collected from surfaces above floor levels using a conventional vacuum cleaner device and nylon dust collecting socks. To eliminate the coarse fraction, dust bag dust samples were size homogenized by filtering through a sterile strainer. Samples were dried in a desiccator prior to aliquoting and stored at 20°C. Sampling point selected in living room	The raw extracts were diluted and analyzed without further cleaning in a mixture of acetonitrile, water, and acetic acid (79/20/1), and the raw extracts were diluted and analyzed without further cleaning	A method for determining multimycotoxins in food and feed was created, and it was expanded to include a list of 159 fungal and 27 bacterial metabolites for the examination of naturally infected indoor samples. Its purpose is to demonstrate the usefulness of a multianalyte LC-MS/MS-based spiking at many levels for determining extraction performance parameters, matrix effects, and recoveries. Since dust samples absorb a substantial quantity of solvent relative to food and feed matrixes, the fraction of extraction solvent has been increased. In order to completely utilize instrument capacity for data gathering, each analyte's availability time was defined in the sMRM mode, where retention time and dwell time were fiercely generated for each point in time from the target scan time, for each analyte	When compared to pure methanol and ethyl acetate, the acidified mixture of acetonitrile and water provided the optimum compromise for extracting chosen metabolites. Compared to mortar, carton-gypsum board and coarse-soil-containing splints, settled house dust caused severe matrix effects and incomplete extraction led to low recoveries of analytes due to complex composition from cell fragments, and many other organic compounds accumulated on particulate matter HPLC-MS-MS and GC-MS-MS have both been shown to be useful analytical methods for detecting some of the most toxic mycotoxins produced by molds that are commonly found in moist indoor environments. These technologies are sensitive that they can identify STRG, VER, and TRID in both mold-affected building materials and house dust	[25, 26]; Bloom et al. 2007; [41]
6	Malaysia	Airborne dust was collected by both the cotton swab and Petri dish. Settled dust samples were collected by swabbing 60 cm^2^ of surface (1 × 60 cm per swab) from the top frame of the blackboard in each classroom. The blackboard top frame was divided into a left and right part, with the left-side dust samples used for fungal DNA analysis and the right-side samples for mycotoxins analysis. Sampling point was selected at the blackboard frame in a classroom	Samples were extracted with methanol, dissolved with DCM, applied to PEI-bonded silica gel column, eluted, evaporated, and redissolved with methanol, and filtered into vials for HPLC injection. For GC-MS injection, methanolic sample extracts are mixed with IS, hydrolyzed and extracted with methanol, and evaporated to dryness. Dried extracts were later derivatized and heated prior to injection	Establishment on GC-MS and GC-MS-MS methods for determination of mycotoxins (verrucarol and trichodermol) and fungal biomass marker (ergosterol) in contaminated indoor environmental samples. Establishment of the LC-MS-MS method for determination of mycotoxins (sterigmatocystin, gliotoxin, aflatoxin B1, and satratoxins G and H) and water-damaged indoor environments samples. Application of different derivative reagents (trimethylsilyl, pentafluorobutyryl, and heptafluorobutyryl) for optimizing determination of mycotoxin with GC-MS and GC-MS-MS. Carryover and ghost peak formation were triggered by adsorption of nonderivatized or semiderivatized mycotoxin in the instrument injector and were overcome by regular injection of a mixture of derivatized reagent with solvent, avoid washing derivatized extracts with water and maximum injection of extracts, to minimize risk of injector contamination	In all samples, mycotoxins were discovered using MSMS and SIM (NICI). Peaks were found in SIM analysis at the correct retention duration but with a significant background noise level due to interference from a partially coeluting chemical. The MSMS analysis, on the other hand, resulted in significantly decreased background noise and higher detection specificity. Heptafluorobutyryl (HFB) derivatives of selected mycotoxins show negligible fragmentation and have good GC-MS and GC-MS-MS detection sensitivity. Combining CI and negative ion (NICI) detection with MS-MS yielded the best detection sensitivity and specificity. Because of its lower detection limit, higher noise reduction, and considerably increased detection specificity, the NICI mode analysis was frequently preferred. HPLC-MS-MS and GC-MS-MS have both been shown to be useful analytical methods for detecting some of the most toxic mycotoxins produced by molds that are commonly found in moist indoor environments. These technologies are so sensitive that they can identify STRG, VER, and TRID in both mold-affected building materials and house dust	[28]; Bloom et al. 2007; [41]
7	Finland, the Netherlands, Spain	Methanol-soaked foam swabs were pushed around the test area, and adherent dust was carried into methanol-filled vials. A few sampling regions are combined to provide a collective sample per site. The samples were collected at ambient temperature, sealed with parafilm, and stored at −20°C until they were analyzed. Classrooms, hallways, teacher's lounges, libraries, dining halls, bath/shower rooms, storage rooms, and other areas of the school building were chosen as sampling points	The methanolic suspensions were shaken using a rotary shaker, allowed to settle and the clear upper methanolic layers were transferred into glass vials equipped with glass microinserts. The diluted raw extract was directly injected into the HPLC-MS/MS instrument. Then, the methanolic samples were evaporated and reconstituted in DCM. The liquids were applied to preconditioned PEI-bonded silica gel columns. The eluates were evaporated and redissolved in methanol. Methanolic materials were combined with IS, evaporated, hydrolyzed, and extracted with DCM and water. The extract was transferred to fresh vials, evaporated, and kept in a desiccator overnight before being derivatized with HFBI and heated before injection into the GC/MS-MS	For the measurement of 186 fungal and bacterial secondary metabolites, an HPLC-MS/MS technique was developed. A QTRAP 4000 LC-MS/MS system with a TurboIonSpray electrospray ionization (ESI) source and an 1100 series HPLC system were used for detection and quantification in the scheduled multiple reaction monitoring (sMRM) mode. Using a GC-triple-quadrupole MS/MS apparatus, establish GC-MS/MS analyses for the determination of mycotoxins (verrucarol and trichodermol). Carryover and ghost peak formation were triggered by adsorption of nonderivatized or semiderivatized mycotoxin in the instrument injector and were overcome by regular injection of a mixture of derivatized reagent with solvent, avoid washing derivatized extracts with water and maximum injection of extracts, to minimize risk of injector contamination	Since matrix-matched calibration was found to be insufficient for correction of these effects in the very heterogeneous dust matrix, HPLC-MS/MS findings were not corrected for incomplete extraction and/or signal suppression/enhancement due to coeluting matrix constituents. The acquisition of two sMRM transitions per analyte was required for positive identification, and the LC retention time and intensity ratio of the two sMRM transitions had to coincide with the associated values of a genuine standard within 0.1 min and 30% rel., respectively. High prevalence of mycotoxins due to high detection sensitivity offered by the triple-quadrupole mass spectrometers in MS-MS mode. HPLC-MS-MS and GC-MS-MS have proven to be complementary analytical tools for detecting some of the most potent mycotoxins produced by molds frequently encountered in damp indoor environments. These methods are so sensitive that STRG, VER, and TRID can be detected not only in mold-affected building materials but also in house dust	[26, 29, 42]
8	Croatia	Using a MAS-100 Eco air sampler (Merck, Darmstadt, Germany) with 400 holes (hole to agar impactor) and dichloran 18 percent glycerol agar (DG18) plates, airborne fungi were collected in two-month intervals at apartments (APs), basements (BS), grain mill (GM), and open air (ODA) over a one-year period	Growth aspergilli were cut at 3 plugs of 6 mm diameter by using cylindrical drill and transferred into Eppendorf tubes containing 1000 *μ*l of solvent mixture methanol-DCM-ethyl acetate with a ratio (1/2/3) supplemented with 1% (v/v) formic acid. The clean extracted sample was sonicated. Ultrasonically extraction was transferred into clean vials using syringe with 0.45 *μ*m filters (Sartorius, Germany). Then, the extracted samples were dried under stream of nitrogen gas before kept at −20°C. The dried samples were redissolved in 500 *μ*l of methanol/water (3/1) mixture and filtered through 0.45 *μ*m filters into new vials. Collected dust samples were extracted with solvent mixture methanol-DCM-ethyl acetate with a ratio (1/2/3) supplemented with 1% formic acid. The sample mixture was shaken for 1 hour at room temperature and centrifuged. The supernatants were collected and evaporated to dryness. Dried residues were redissolved with ration of 5 mL of methanol/water (3/1). All samples were filtered through 0.45 *μ*m filters into clean vials	Analysis by using ClassVP 6.2 software and control DGU-14A vacuum degasser, a quaternary LC-10ADVp pump, a CTO-10ASVp column thermostat, an SPD-10ADVp UV-VIS detector, and an SCL-10 system (HPLC Shimadzu). Separation was performed on a LiChroCART Purosphere STAR RP-18 250 mm × 4 mm column with 5 *μ*m particle size (Merck, Hungary) coupled with a Lichospher 100 RP-18 guard column (Merck, Hungary) at 35°C. Mobile phase with setting of flow rate of 0.5 mL/min, and the injection volume was 20 *μ*L. The gradient elution was achieved by changing the ratio of methanol and water. STC was quantified by measuring peak areas in an HPLC chromatogram and comparing them to STC calibration standards	Sharp peak of less polar metabolite eluted right before STC in *A. jensenii* and *A. venenatus* chromatograms was discovered. This study did not analyze it by LC/MS/MS analyses and considering STC-derived metabolites and/or precursors belonging to 5-methoxy-STC. Another research had compared with this study and found the metabolite detected by HPLC-DAD and TLC with AlCl_3_	[30]
9	Poland	Moldy surfaces in form of scrapings and airborne dust from 22 moldy dwellings in winter season	All filters were washed with 4 mL of a solvent ratio of acetonitrile/water/acetic acid (79/20/1). Then, the samples were extracted for 90 min with KL 2 Multipurpose Shaker (Edmund Bühler GmbH, Germany). The samples were centrifuged at 1000*g* for 15 min. The supernatant was dried under gentle stream of nitrogen at 40°C. 1 mL of acetonitrile was dissolved into dried residue	HPLC analysis was conducted using a symmetry C18 column (150 × 2.1 mm 5 *μ*m) with column temperature set at 40°C and 10 *μ*L of sample injection. Gradient elution using mixture of methanol: acetate buffer 0.1 mol/L pH 4.6 serves as mobile phase. Simultaneous determination was performed at an absorbance of 247 nm and 326 nm. UV spectra at an absorbance of 210–500 nm and retention time of the standards were used to analyze the samples	The method collection like scraping and airborne dust did not indicate quantities exceeding the limit of determination of the method. However, the study revealed that ST and RC were present in the fungi cultivated from scrapings. This demonstrates that toxinogenic strains of *Aspergillus versicolor* and *Penicillium chrysogenum* display this feature when cultivated on MEA medium in the laboratory. All the studies, including this study, used the HPLC approach; therefore, it is unclear why this study was not able to demonstrate the presence of mycotoxins in the air dust and scrape samples from flats	[27]
10	Malaysia	Settled dust samples were collected by swabbing 60 cm^2^ of surface (1 × 60 cm) from the top frame of the blackboard in each classroom. The blackboard top frame was divided into a left and right part, with the left-side dust samples used for fungal DNA analysis and the right-side samples for mycotoxin analysis. Sampling point was selected at the blackboard frame in a classroom	Three types of liquid extraction were performed. First, extraction with methanol with three different samples in which the pieces of agar (approximately 5 cm^2^), dust sample (∼0.4 g), and building material (0.3 to 3 g) were immersed with methanol at room temperature for 72 h. The sample was then centrifuged for 5 min at 3,200 rpm, and the supernatant was poured into new tubes. Second, extraction with heptane 100 *μ*L of sterile water added and the mixture were extracted twice with 2 mL of heptane. Finally, methanolic phases were dissolved in DCM and applied to PEI (1 mL bonded silica gel columns) after being evaporated under a moderate stream of nitrogen. The PEI column already preconditioning the columns with 4 mL of methanol and DCM before eluted with sample. Then, 5 mL of DCM was eluted into the sample through the PEI column and had been evaporated under nitrogen. Prior to analysis, the sample was redissolved with 1 mL of methanol and filtered through 0.45 *μ*m Millex syringe filters. The methodology was modified by the previously mentioned method [41]	HPLC-MS analysis was performed using a ProStar HPLC/1200L triple-quadrupole MS-MS system (Varian Inc., Walnut Creek, CA). 20 *μ*L of each sample was injected, using an autosampler (model 410; Varian), into a Polaris 5 *μ*M C18-A 150 by 2.0 mm RP-18 column equipped with a MetaGuard 2.0 mm Polaris 5-*μ*M C18-A precolumn (Varian). Reserpine was used as the internal standard. GC-MS analysis sample was performed on a CP-3800 gas chromatograph equipped with a fused-silica capillary column (FactorFOUR™, VF-5 ms, 30 m × 0.25 mm i.d., 0.25 mm film thickness) and connected to a 1200 L triple-quadrupole MS-MS detector (Varian Inc., Walnut Creek, CA, USA). Derivatives were analyzed in both EI modes, at an energy of 70 eV and an ion source temperature of 250°C (TMS derivatives) or 200°C (HFB derivatives) and in NICI mode with methane as ionization gas at a pressure of 0.8 kPa and a source temperature of 200°C. Volumes of 1-2 mL were injected in the splitless mode with a helium carrier gas pressure of 69 kPa, using a CombiPAL autosampler (CTC Analytics AG, Zwingen, Switzerland). The MS-MS conditions were optimized by repeatedly injecting 0.1–1 ng amounts of standards at different collision energy, ion source temperature, and argon pressure in the collision cell. The parameters that gave the largest product ion peak area were selected	A supplement of 10 mM ammonium acetate and 20 *μ*M sodium acetate was added to the methanol aqueous buffer to increase the cationization in the electrospray ionization mode. Ten microliters of methanol was injected in between samples to minimize cross-contamination. A mix of HFBI and acetone (1/3) was injected in between samples to eliminate any trace of underivatized or semiderivatized VER/TRID	[40], Bloom et al. 2007, [41]
11	France	Aerosolization of produced mycotoxins from contaminated wallpapers. Sampling point was selected at wallpapers	Four MCT (SG, SH, VerJ, and RL2), MPA, and STC were extracted from samples (wallpaper and fiberglass disks) by gentle mechanical agitation on an agitation table in chloroform-methanol (2/1). Mycophenolic acid-d3 and o-methyl sterigmatocystin were added at known concentrations before starting extraction in order to serve as internal standards for MPA and STC, respectively. For MCT, verrucarin A was chosen as an internal standard, as already described. After 4 h, extracts were centrifuged for 5 min at 3,500 rpm and passed through a phase separator (PS) filter (Whatman 1 PS). The filtered extracts were evaporated to dryness and suspended in 1 mL of methanol	Quantification was performed using an Acquity ultraperformance liquid chromatography (UPLC) system coupled to Xevo triple-quadrupole mass spectrometer ethanol. The desolvation temperature and nitrogen flow rate were set at 650°C and 800 liters/h, respectively. Argon was used as the collision gas at a flow rate of 0.12 mL/min. Mycotoxins (5 *μ*L of samples) were eluted on an Acquity BEH C18 column (2.1 by 100 mm, 1.7 m; Waters), with ACN-H_2_O gradient (0 to 0.5 min), 10% ACN; (0.5 to 4 min), 90% ACN) at a flow rate of 0.35 mL/min. Quantification was carried out by multiple reaction monitoring (MRM) mode in positive electrospray ionization (ESI). Chromatographic data were monitored using the MassLynx 4.1 software	This study showed that three different toxinogenic species produce mycotoxins during their development on wallpaper. These toxins can subsequently be aerosolized, at least partly, from moldy material. This transfer to air requires air velocities that can be encountered under real-life conditions in buildings. Most of the aerosolized toxic load is found in particles whose size corresponds to spores or mycelium fragments. However, some toxins were also found on particles smaller than spores that are easily respirable and can deeply penetrate the human respiratory tract. All of these data are important for risk assessment related to fungal contamination of indoor environments	[32]
12	France	Dust was collected in the air using a CIP 10 sampler (Tecora, France) with an inhalable fraction selector	Mycotoxins were extracted from the porous polyurethane foam filter (PUF) using methanol, and the PUF capsules were rinsed to remove any dust particles	High-resolution mass spectrometry combined with ultrahigh-performance liquid chromatography (UPLC-Q-Orbitrap HRMS). A BEH C18 column (2.1 mm, 10 mm, 1.7 mm) and precolumn were used to separate the analytes (Waters). In positive electrospray ionization mode, mass spectrometry detection was performed using a Q Exactive mass spectrometer (Thermo Fisher Scientific™). Analytes were detected by comparing product ion retention durations and mass accuracy	Three waste management sectors were incorporated in the sample strategy, as well as both stationary and personal air samplings. The use of UPLC-HRMS for quantitative analysis of airborne mycotoxins is a very sensitive and specific technology that allowed for the detection of low mycotoxin concentrations in the air. The application of measuring results to the assessment of health risks	[23]
13	Germany	Samples were collected using a vacuum cleaner from different waste management units and houses inhabited by less than 5 people	Headspace volatile extraction procedure fully automated by an autosampler for microbial volatile organic compound using Agilent 6890 GC QTRAP 4000 LC-MS/MS with C18 column	Method for LC-MS-MS was adopted from [26]	The use of LC-MS-MS and GC-MS provides many microbial metabolite and volatile anthropogenic chemical presence in indoor environments. This is the first study to compare individual settled floor dust samples derived from relatively different indoor environments using both LC-MS/MS and GC-MS methods	Vishwanath et al. 2011 [26]

**Table 4:** Mycotoxin analysis using GC-MS-MS/LC-MS-MS.

No.	Analyte/metabolite/mycotoxin	System/instrumentation/method	Validation			Reference
			Precision/accuracy	LOD/LOQ/CV	Recoveries/*R*^2^	
1	Fungal aerosols AFB1, B2, G1, G2, M1, diacetoxyscirpenol, gliotoxin, mycophenolic acid, neosolaniol, ochratoxin A, T-2 toxin (method 1); alternariol, deoxynivalenol, deepoxy-deoxynivalenol, 3-acetyldeoxynivalenol, 15-acetyldeoxynivalenol, fusarenon X, HT-2 toxin, verrucarol, zearalenone (method 2)	HPLC-MS/MS, MRM, positive and negative modes, ESI		Quantification limit (ng/filter): 0.05–0.50	Recovery: 56–101.10%	[21]

2	DON, AFB1, OTA, toxin T-2 (T-2), zearalenone (ZEA), and sterigmatocystin (STE)	HPLC/ESI-MS/MS, MRM, positive and negative modes, ESI, and the ion source temperature of 150°C in splitless mode	The intraday precision: 4% to 8%. The interday precision: 4% to 14%	LOD in the matrix: 0.8 (AFB1) to 8.5 ng·mL^−1^ (T-2 toxin) LOQ in the matrix: 3.2 (AFB1) to 26 ng·mL^−1^ (T-2 toxin)	Recoveries of all tested mycotoxins (72–95%) with a CV% below 10%. when the matrix-matched standard curve without the ISTD was used	[39]

3	Verrucarol	GC-MS/MS, negative chemical ionization mode using methane as ionization gas at the energy of 150 eV	NIL	NIL	Recovery of verrucarol was 94%	[24]

4	3 mycotoxins (HT-2 toxin, nivalenol, patulin) (method 1); 14 mycotoxins (aflatoxins B1, B2, G1, G2, M1, citrinin, diacetoxyscirpenol, fumagillin, gliotoxin, mycophenolic acid, neosolaniol, ochratoxin A, T-2 toxin, verruculogen) (method 2)	HPLC-MS/MS, MRM, positive and negative modes, ESI	NIL	Quantification limit (ng/filter) ranged from 0.05 to 23.49	Recoveries ranged from 42% (HT-2) to 97% (DAS)	[22]

5	A total of 186 compounds comprise 159 fungal metabolites and 27 bacterial metabolites	Liquid chromatography/tandem mass spectrometry (LC-MS/MS), specifically QTRAP 4000 LC-MS/MS equipped with a TurboIonSpray electrospray ionization source and an 1100 series HPLC system	Positive identification was obtained by the acquisition of two sMRM transitions per analyte that made 4.0 identification points according to decision by 2002/657/EC. The LC RT and the intensity ratio of the two sMRM transitions had to agree with the related values of an external standard within 0.1 min and 30% relative, respectively	Generally, in the low *μ*/Kg range and exceeded 50 *μ*/kg only for those compounds exhibiting a low apparent recovery or a general low MS/MS sensitivity	29/36 analytes (81%) tested reached within a target range of 70–120%	[25, 26]; [41]; [42]

6	Aflatoxin B1, gliotoxin, satratoxin G, satratoxin H, and sterigmatocystin Trichodermol and verrucarol	HPLC-MS. A ProStar HPLC/1200L triple-quadrupole MS-MS system was used, 5 *μ*M C18-A 150 by 2.0 mm RP-18 column equipped with a MetaGuard 2.0-mm 5 *μ*M C18-A precolumn. Reserpine was used as the internal standard GC-MS-MS using a CP-3800 GC-triple-quadrupole MS-MS system. The derivatives were analyzed by using MS-MS in negative ion chemical ionization mode, at an energy of 70 eV and an ion source temperature of 150°C, and with ammonia as the ionization gas	The electrospray ionization MS parameters achieved maximal detection sensitivity for each standard when their spectrum showed prominent parent and product ions	Trichodermol and verrucarol 6 pg, sterigmatocystin 12 pg, and aflatoxin B1 and gliotoxin 125 pg Satratoxins G and H were not quantified	The recovery value was 53 ± 6% with 11.2% CV	[28, 42–43]

7	A total of 186 compounds comprise 159 fungal metabolites and 27 bacterial metabolites Trichodermol & verrucarol	Liquid chromatography/tandem mass spectrometry (LC-MS/MS), specifically QTRAP 4000 LC-MS/MS equipped with a TurboIonSpray electrospray ionization source (ESI) and an 1100 series HPLC system. Elution was carried out in binary gradient mode GC-triple-quadrupole MS/MS instrument	Positive identification was obtained by the acquisition of two sMRM transitions per analyte that made 4.0 identification points according to decision by 2002/657/EC. The MSMS conditions were optimized by repeatedly injecting 0.1–1 ng amounts of standards at different collision energy, ion source temperature, and argon pressure in the collision cell. The parameters that gave the largest product ion peak area were selected. Detection sensitivity, defined as amounts of standards injected with a signal-to-noise ratio >4	Generally, in the low *μ*/kg range and exceeded 50 *μ*/kg only for those compounds exhibiting a low apparent recovery or a general low MS/MS sensitivity	29/36 analytes (81%) tested reached within a target range of 70–120%	[26, 29, 42, 43]

8	Airborne sterigmatocystin STC	HPLC/UC-VIS	NIL	Quantification limit (0.025–250.0 *μ*g/ml)	*R* ^2^ values: 0.9999	[30]

9	Sterigmatocystin roquefortine C	HPLC Waters analytical column	NIL	Limit of determination of the method: 0.2 *μ*g/ml Quantification limit: 0.2–10 *μ*g/ml	NIL	[26]

10	HPLC-MS-MS Aflatoxin B Gliotoxin Satratoxin G Satratoxin H Sterigmatocystin GC-MS-MS Trichodermol Verrucarol	HPLC-MSMS (HPLC/1200L triple-quadrupole MS-MS system). The capillary temperature was 310°C, the capillary voltage was 40 V, the needle voltage was 5,000 V, and the electron multiplier voltage was 2,000 V. The MS spectra were collected as centroid data from m/z 100 to 800, with a scan time of 0.5 s and a scan width of 0.7 s GC-MS-MS (CP-3800): the derivatives were analyzed by using MS-MS in negative ion chemical ionization mode, at an energy of 70 eV and an ion source temperature of 150°C, and with ammonia as the ionization gas (0.4 kPa)	NIL	LOD: 6 pg Trichodermol and verrucarol 12 pg sterigmatocystin 125 pg aflatoxin B and gliotoxins	NIL	[40]

11	Satratoxin G (SG), satratoxin H (SH), verrucarin J (VerJ), roridin L2 (RL2), mycophenolic acid (MPA), and sterigmatocystin (STC)	UPLC coupled to a Xevo triple-quadrupole mass spectrometer. Quantification was carried out by multiple reaction monitoring (MRM) mode in positive electrospray ionization (ESI)	NIL	LOD: (1) MPA, STC = 0.2 ng/mL (2) RL2 = 0.2 ng/mL (3) VerJ = 5 ng/mL (4) SH, SG = 10 ng/mL LOQ: (1) MPA, STC, RL2, VerJ = 10 ng/mL (2) SG, SH = 100 ng/mL	NIL	[32]

12	Satratoxin H Satratoxin G Roridin L2 Roridin E Atranone A Atranone B Dolabellane Stachybotrylactam Stachybotrylactam (isomer) Stachybotryamide Stachybotrydial Mer-NF-5003-B Trichodermin	UHPLC-QTOF to UHPLC-QqQ	NIL	LOD and LOQ (ng/cm^2^), respectively: Satratoxin H (15, 50) Satratoxin G (15, 50) Roridin L2 (0.1, 0.2) Roridin E (0.1, 0.2) Atranone A (2, 6) Atranone B (2, 6) Dolabellane (2, 6) Stachybotrylactam (2, 6) Stachybotrylactam isomer (2, 6) Stachybotryamide (2, 6) Stachybotrydial (2, 6) Mer-NF-5003-B (2, 6) Trichodermin (5, 17)		[31]

13	Aflatoxin B1 Sterigmatocystin Gliotoxin Ochratoxin	Dionex Ultimate 3000 UPHLC mass spectrometry detection was carried out on a Q Exactive mass spectrometer (Thermo Fisher Scientific™) operated in positive electrospray ionization (ESI (+)) mode	NIL	Mass spectrometry LOQ 0.1 ng·mL^−1^ (i) Aflatoxin B1 (ii) Gliotoxin (iii) Sterigmatocystin 0.2 ng·mL^−1^ (i) Ochratoxin A		[23]

14	71 volatile organic compounds 18 microbial volatile organic compounds	Agilent 6890 GC QTRAP 4000 LC-MS/MS with C18 column	NIL	3 *μ*g/kg for (i) Sterigmatocystin	Less than 50%	Vishwanath et al. 2011 [26]
